# Nucleotide metabolism and cervical cancer: A comprehensive study of molecular subtypes and therapeutic targets

**DOI:** 10.1097/MD.0000000000051463

**Published:** 2026-11-06

**Authors:** Feng Li, Chaoen Bao, Yinliang Liu, Bing Liu, Fushan Zhai

**Affiliations:** a Department of Oncology, Hebei Medical University Third Hospital, Shijiazhuang, Hebei, China.

**Keywords:** bioinformatics, cervical cancer, NMF, nucleotide metabolism, prognostic model

## Abstract

Cervical cancer (CC) is among the most prevalent malignancies in women worldwide. Tumor progression involves profound metabolic reprogramming, in which nucleotide metabolism sustains deoxyribonucleic acid/ribonucleic acid synthesis and proliferation; however, its role in CC remains unclear. Transcriptomic and clinical data of CC patients were obtained from The Cancer Genome Atlas. Nonnegative matrix factorization classified patients into molecular subtypes based on nucleotide metabolism-related genes and compared their survival, immune infiltration, somatic mutations, and drug sensitivity. Mfuzz soft clustering identified subtype-associated expression patterns. Gene set variation analysis scoring, differential expression analysis, and Gene Ontology/Kyoto Encyclopedia of Genes and Genomes enrichment of differentially expressed genes were then performed. Prognostic models were constructed using multiple machine learning algorithms, and the model with the highest concordance index (C-index) was selected. Patients were stratified into high- and low-risk groups and assessed using multivariate Cox regression, Kaplan–Meier, and time-dependent receiver operating characteristic analyses. Immune checkpoint inhibitor response was predicted using the tumor immune dysfunction and exclusion algorithm, and molecular docking was performed between core genes and candidate compounds. Nonnegative matrix factorization revealed 5 subtypes with distinct outcomes: cluster 2 showed the worst prognosis, whereas clusters 3 and 4 had favorable survival. *TTN*, *KMT2C*, *HUWE1*, and *PIK3CA* were frequently mutated across subtypes. B cells, regulatory T cells, CD8+ T cells, and M2 macrophages were enriched in clusters 3 and 4. VX-680, CIL70, and BRD-K01737880 exhibited significantly lower IC50 values in the poor-prognosis group. Seven key genes (*SERPINE1*, *CAV1*, *SOD2*, *SLC16A1*, *XDH*, *CYP27B1*, and *NAGS*) were identified by Mfuzz clustering. Forty-two differentially expressed genes between the high- and low-score groups were predominantly enriched in xenobiotic response, nutrient sensing, small-molecule catabolism, inflammatory response, tumor necrosis factor signaling, apoptosis, and hypoxia. The generalized boosted regression model achieved the highest C-index, with 1-, 3-, and 5-year area under the curves of 0.62, 0.67, and 0.61, indicating modest discrimination. Tumor immune dysfunction and exclusion analysis indicated reduced immune checkpoint inhibitor responsiveness in the high-risk group. Molecular docking predicted favorable binding between core genes and candidate compounds, providing hypotheses for experimental validation. This study delineates the associations of nucleotide metabolism-related genes with molecular classification and prognosis in CC and identifies potential biomarkers, candidate therapeutic agents, and a prognostic model providing complementary assessment value.

## 1. Introduction

Cervical cancer (CC) is among the most prevalent malignancies affecting women globally and has become a major public health concern.^[1]^ In 2018, approximately 570,000 new cases and 310,000 deaths were reported worldwide.^[2]^ The etiology of CC is multifactorial, with persistent human papillomavirus infection recognized as the principal cause, along with contributing factors such as reproductive history and tobacco use.^[3]^ Current therapeutic approaches primarily include surgery and radiotherapy, often combined with chemotherapy.^[4]^ While early-stage CC is largely curable, advanced disease is associated with poor prognosis and limited survival. The 5-year survival rate exceeds 90% for early-stage patients but drops to 5% to 15% in advanced stages.^[5,6]^ Consequently, identifying the key genes involved in CC progression is essential for improving early diagnosis, patient outcomes, and overall prognosis.

A fundamental feature of cancer metabolism is the dysregulation of nucleotide synthesis and utilization.^[7]^ Nucleotides support deoxyribonucleic acid (DNA) and ribonucleic acid synthesis and are therefore essential for rapid cellular proliferation.^[8]^ Altered nucleotide metabolism has also been associated with treatment resistance, immune evasion, and metastasis.^[9,10]^ In hepatocellular carcinoma, nucleotide metabolic reprogramming has been linked to the regulation of immune-checkpoint expression.^[11]^ Although inhibitors of nucleotide metabolism have shown antitumor activity in preclinical models, the biological and clinical relevance of this pathway in CC remains incompletely understood.

In this study, we analyzed transcriptomic and clinical data from The Cancer Genome Atlas cervical squamous cell carcinoma and endocervical adenocarcinoma (TCGA-CESC) cohort to identify nucleotide metabolism-related molecular subtypes and characterize their prognostic, genomic, and immune features. We subsequently selected subtype-associated core genes and constructed a prognostic model using multiple machine learning algorithms. Predicted responses to immune checkpoint inhibitor (ICI) therapy and potential interactions between core proteins and candidate compounds were also explored. This integrated analysis aimed to clarify the prognostic relevance of nucleotide metabolism in CC and provide a basis for further biological investigation.

## 2. Materials and methods

### 2.1. Datasets download

The expression profiles of CC patients were obtained from the TCGA database and normalized using transcripts per million. The TCGA-CESC cohort included 306 tumor samples of cervical squamous cell carcinoma and endocervical adenocarcinoma, along with 13 normal tissue samples.^[12]^ Genes related to nucleotide metabolism were extracted from the gene set enrichment analysis (GSEA) database (https://www.gsea-msigdb.org/gsea/index.jsp).

### 2.2. CC subtype identification

Based on these 150 genes related to nucleotide metabolism, we performed nonnegative matrix factorization (NMF) clustering analysis on the TCGA-CESC cohort using the “NMF” package.^[13]^ Unsupervised NMF clustering was conducted with the “brunet” method and 100 runs (nruns = 100), and the optimal number of clusters (*K* value) was determined as 5 according to the cophenetic correlation coefficient. Kaplan–Meier survival curves for the CC subgroups were generated using the “survival” package.^[14]^

### 2.3. Tumor mutation burden analysis

To characterize genomic mutation differences among CC subtypes, somatic mutation data from the TCGA-CESC dataset were analyzed using the “Maftools” package.^[15]^ Somatic variants were characterized according to variant classification, variant type, and single-nucleotide variant (SNV) class. The somaticInteractions function of the “Maftools” package was applied to identify co-occurring and mutually exclusive mutations across subtypes. Furthermore, genomic instability metrics including aneuploidy score, homologous recombination deficiency score, fraction of genome altered, copy number segment count, and non-silent mutation rate were calculated to comprehensively assess genomic variation among subtypes.

### 2.4. Analysis of immune cell infiltration

QuanTIseq is an immune deconvolution algorithm designed to estimate immune cell infiltration levels.^[16]^ Normalized gene expression data, expressed as transcripts per million, from the 5 tumor clusters identified in the TCGA-CESC cohort were used as input. The “quantiseqr” package was subsequently applied to calculate the relative proportions of various immune cell types via deconvolution analysis.

### 2.5. Mfuzz-based clustering of subtype-associated expression patterns

To characterize subtype-associated expression patterns of nucleotide metabolism-related genes, we applied Mfuzz soft clustering in the “Mfuzz” package^[17]^ to the mean expression value of each gene within each NMF-defined subtype. The subtype-level expression values were entered in the original NMF cluster-label order (clusters 1–5). This ordering was used solely to organize the input matrix and does not represent longitudinal sampling, repeated measurements, temporal progression, or a biological transition between subtypes. Nine fuzzy clusters (*k* = 9) were generated. The cluster whose subtype-specific expression pattern showed the closest concordance with the observed prognostic differences among the NMF-defined subtypes was selected, and its constituent genes were retained for downstream analysis. Core genes within the optimal cluster were visualized using the “ggplot2” package.^[18]^

### 2.6. Drug sensitivity analysis

The “oncoPredict” package^[19]^ was employed to estimate half-maximal inhibitory concentration (IC50) values of various chemotherapeutic agents across the 5 clusters identified via NMF analysis. The results were visualized using heatmaps. Drugs showing significant differences between the cluster with the most favorable prognosis and the cluster with the poorest prognosis were identified using thresholds of |log_2_ fold change| > 1.5 and adjusted *P* < .05.

### 2.7. Molecular docking

The 3D structures of small molecules were retrieved from PubChem and converted to Protein Data Bank (PDB) format using PyMOL (Schrödinger, LLC). Corresponding PDB structures of core genes from the optimal cluster in TCGA-CESC were obtained from the PDB. Small molecules were prepared in AutoDock Vina by removing hydrogens and analyzing rotatable bonds, while protein structures were preprocessed by removing water molecules and adding all hydrogens. Docking grids were defined, binding energies were calculated, and hydrogen bond interactions were assessed. Docking results were visualized using PyMOL.

### 2.8. Differential expression analysis

Gene set variation analysis (GSVA) was employed to calculate the enrichment scores of the core genes derived from the selected Mfuzz cluster in each TCGA-CESC CC sample. All samples were stratified into high- and low-score groups according to the median enrichment score. Differentially expressed genes (DEGs) between the 2 groups were identified using the “limma” package,^[20]^ with screening criteria set at |log_2_ fold change| > 1 and *P* < .05.

### 2.9. Functional enrichment analysis

To elucidate the biological functions of the DEGs, Gene Ontology (GO) and Kyoto Encyclopedia of Genes and Genomes (KEGG) pathway enrichment analyses were performed using the “clusterProfiler” package.^[21]^ GO terms and KEGG pathways with *P* < .05 were regarded as statistically significant, and the enrichment results were visualized with the “ggplot2” package. GSEA was also conducted using “clusterProfiler,” with “c2.cp.kegg.v7.0. symbols.gmt” as the reference gene set and a significance threshold of *P* < .05.

### 2.10. Construction of a prognostic model for CC patients

The 5 clusters identified through the NMF analysis were used as the data foundation for constructing the prognostic model. Multiple machine learning algorithms were applied, including Cox proportional hazards regression with boosting, generalized boosted regression model (GBM), partial least squares cox regression, random survival forest, survival support vector machine, elastic net (α = 0.1), lasso regression, ridge regression, and supervised principal component analysis. All models were optimized using 10-fold cross-validation on the training set, and Harrell’s concordance index (C-index) was calculated on an independent validation set to evaluate the predictive performance.

Subsequently, in the GBM model, the risk score was calculated from the expression levels of core genes in the selected Mfuzz cluster within the TCGA-CESC samples. CC patients were then stratified into high- and low-risk groups based on their risk scores. The independent prognostic value of the risk score for overall survival was evaluated using a multivariate Cox regression analysis. Receiver operating characteristic (ROC) curve analysis was performed with the “survivalROC” package^[22]^ to assess 1-, 3-, and 5-year predictive performance using the Kaplan–Meier method.

### 2.11. Prediction of immunotherapy response

The tumor immune dysfunction and exclusion (TIDE) algorithm is a computational tool that leverages gene expression profiles to predict responses to immune checkpoint inhibitors.^[23]^ We applied TIDE to assess the potential immunotherapy response in CC patients of the high- and low-risk groups.

### 2.12. Statistical analysis

All bioinformatics analyses were conducted using R software (version 4.2.0; R Foundation for Statistical Computing, Vienna, Austria, http://www.R-project.org). Statistical significance was defined as a *P* value or an adjusted *P* value <.05.

### 2.13. Ethics approval

This study used publicly available de-identified data from the TCGA database, and no ethical approval or informed consent was required.

## 3. Results

### 3.1. Identification of subtypes in TCGA-CESC

To identify the molecular subtypes of CC patients, gene expression profiles from the TCGA-CESC dataset were analyzed using NMF. The optimal number of clusters was determined to be 5 based on the cophenetic coefficient, dispersion, silhouette width, and sparseness (Fig. 1A and B). The corresponding sample sizes for the 5 clusters were 73, 64, 34, 20, and 115, respectively. Kaplan–Meier survival analysis was subsequently performed, revealing significant prognostic differences between patients in clusters 3 and 4 compared with those in cluster 2 (Fig. 1C).

**Figure 1.**
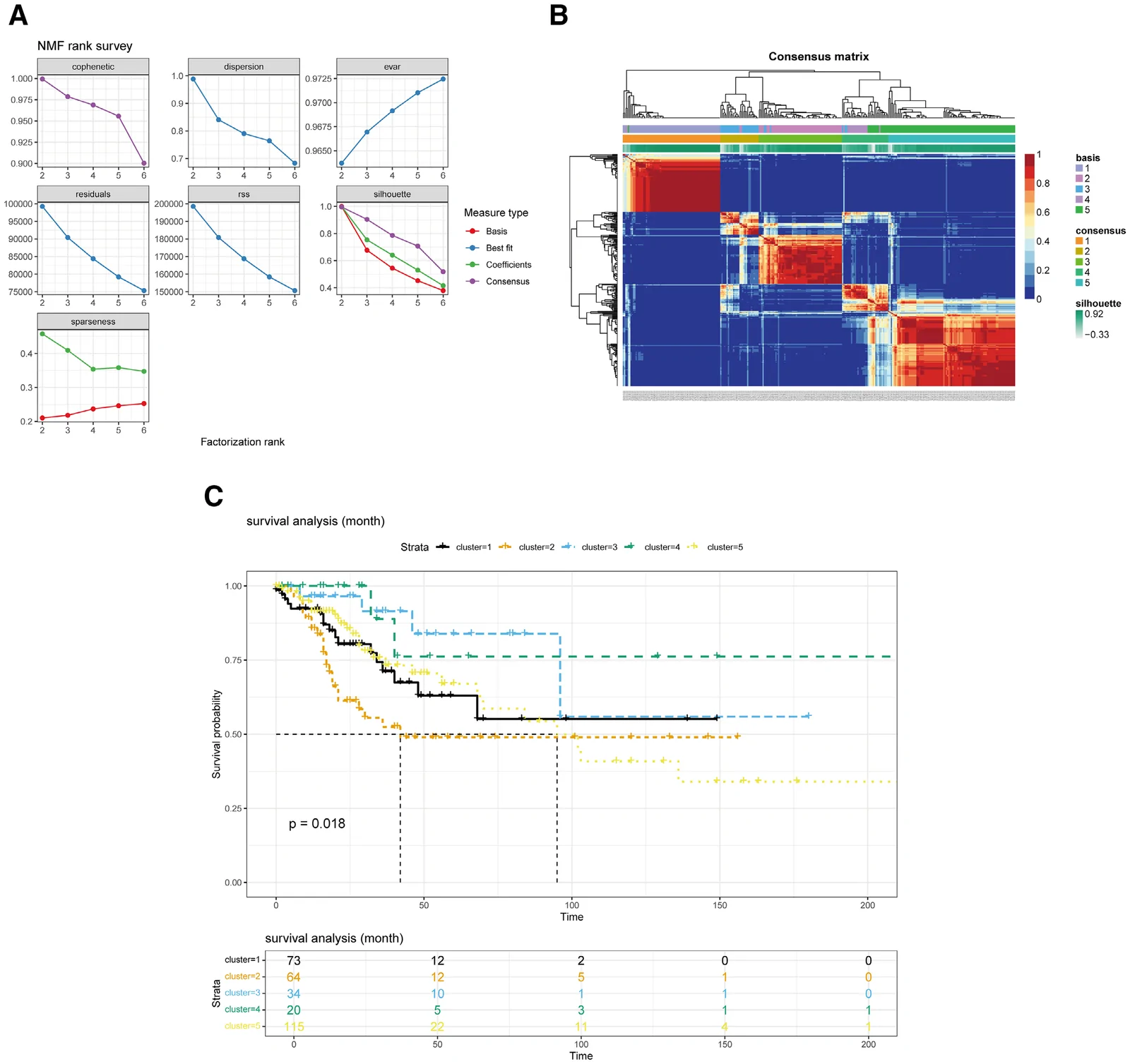
Differences in prognosis among different CC subtypes. (A) The process of NMF subtype clustering. (B) The heatmap of different subtypes. (C) Kaplan–Meier survival analysis between different subtypes. CC = cervical cancer, NMF = nonnegative matrix factorization.

### 3.2. Somatic mutation analysis between different TCGA-CESC subtypes

To investigate the differences in somatic mutations among CC patient clusters, mutation data from the TCGA-CESC dataset were analyzed. Missense mutations were found to be predominant in this cohort. Among the SNVs, C > T and C > A transitions were the most common (Fig. 2A). In cluster 2, 52 of 61 samples (85.25%) exhibited somatic mutations; in cluster 3, 29 of 33 samples (87.88%) harbored somatic mutations; and in cluster 4, 16 of 18 samples (88.89%) showed somatic mutations (Fig. 2B). *TTN*, *KMT2C*, *HUWE1*, and *PIK3CA* exhibited high mutation frequencies across these clusters (Fig. 2B).

**Figure 2.**
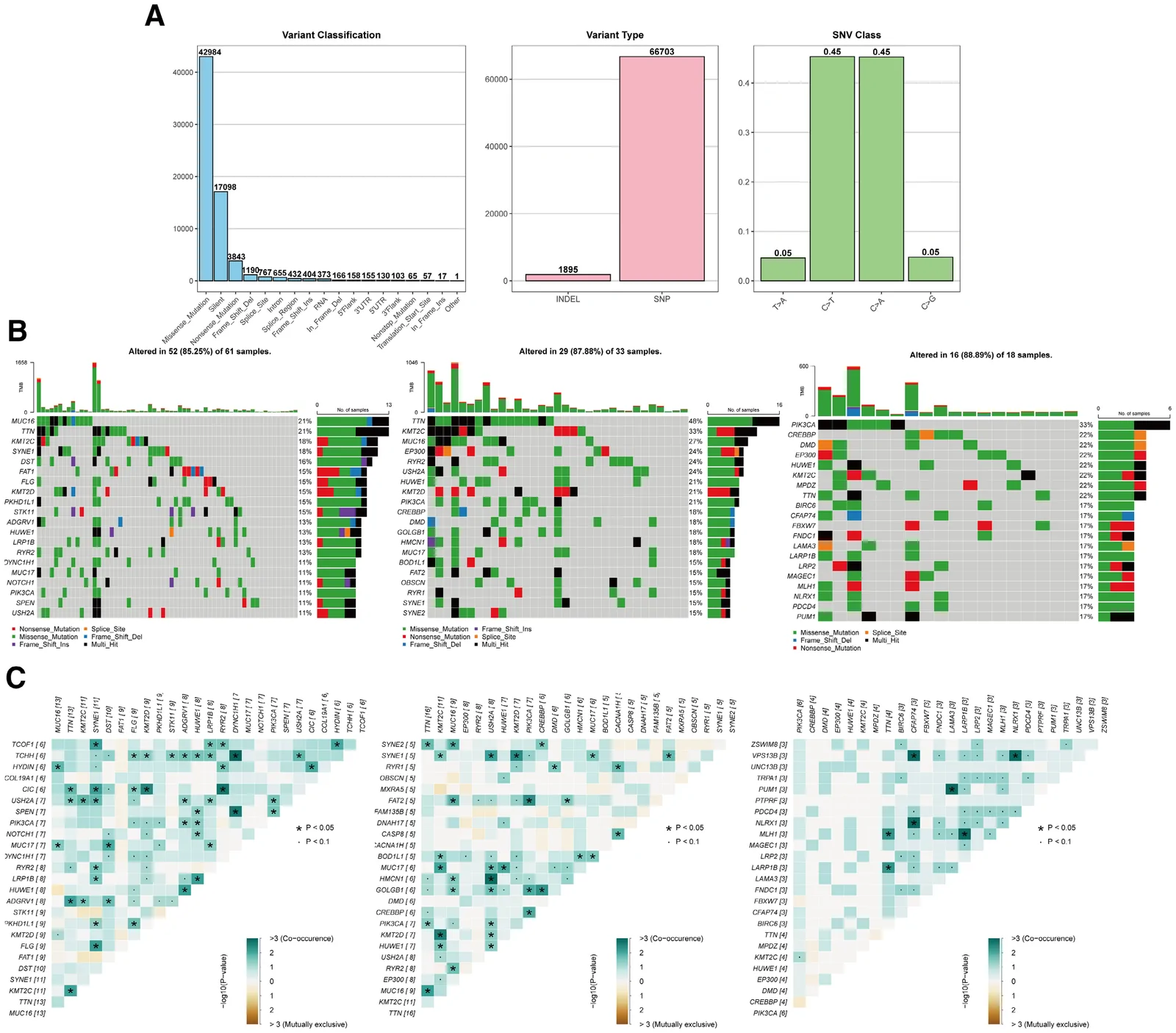
Somatic mutation analysis among different CC subtypes. (A) Overview of the mutation landscape in CC patients. (B) TMB difference in cluster 2, cluster 3, and cluster 4. (C) Correlation analysis between mutated genes in cluster 2, cluster 3, and cluster 4. * represents *P* value <.05. CC = cervical cancer, INDEL = insertion/deletion, SNP = single-nucleotide polymorphism, SNV = single-nucleotide variant, TMB = tumor mutation burden.

Next, we further analyzed the co-occurrence of mutated genes. In cluster 2, *MUC16* mutations significantly co-occurred with *HYDIN* and *MUC17* mutations, whereas in cluster 3, *TTN* mutations were significantly associated with *SYNE2*, *PIK3CA*, and *MUC16* mutations. No significant co-occurrence involving *PIK3CA* mutations was observed in cluster 4 (Fig. 2C).

### 3.3. Differences in immune cell infiltration among TCGA-CESC subtypes

Solid tumors are frequently infiltrated by immune cells that play critical roles in modulating tumor progression.^[24]^ Accordingly, we analyzed immune cell infiltration across different CC subtypes. Compared to cluster 2, clusters 3 and 4 displayed higher levels of B cells, regulatory T cells (Tregs), cluster of differentiation 8 (CD8^+^) T cells, and M2 macrophages (Fig. 3A). Next, we evaluated the tumor purity, immune score, stromal score, and Estimation of STromal and Immune cells in MAlignant Tumors using Expression data score among the subtypes. Relative to cluster 2, clusters 3 and 4 exhibited reduced tumor purity but elevated immune, stromal, and Estimation of STromal and Immune cells in MAlignant Tumors using Expression data scores. Furthermore, the expression profiles of the immune checkpoint/immunoregulatory genes and oncogenes were examined (Fig. 3B). Compared with cluster 2, clusters 3 and 4 showed upregulated expression of immune checkpoint/immunoregulatory genes (*CD274*, *CTLA4*, *HAVCR2*, *IDO1*, *LAG3*, *PDCD1*, and *TIGIT*), while the oncogene *PIM3* was downregulated (Fig. 3C).

**Figure 3.**
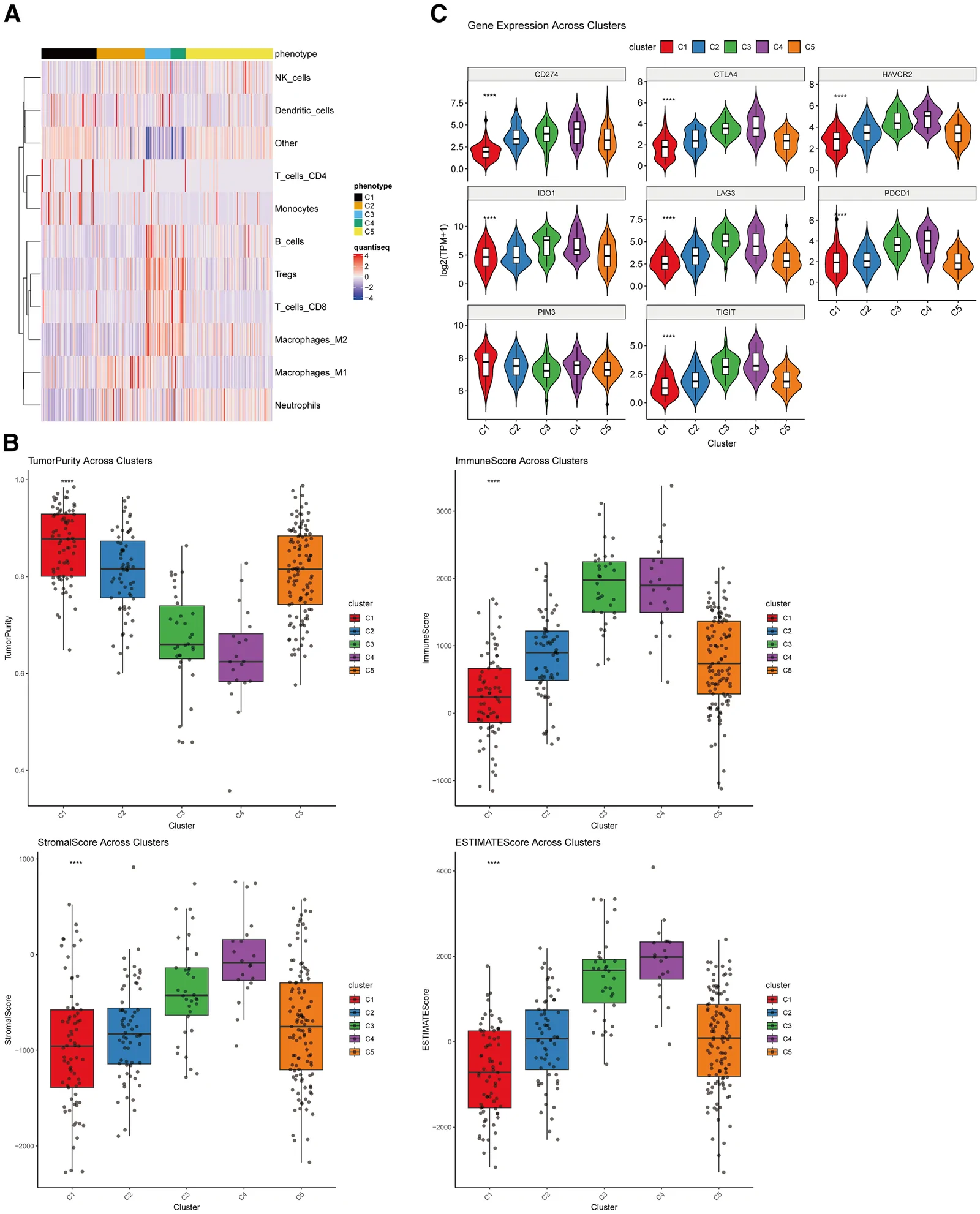
Immune cell infiltration analysis among different CC subtypes. (A) Differences in immune cell infiltration among 5 CC subtypes. (B) Differences in tumor purity, immune score, stromal score, and ESTIMATE score among 5 CC subtypes. (C) The expression of *CD274*, *CTLA4*, *HAVCR2*, *IDO1*, *LAG3*, *PDCD1*, *PIM3*, and *TIGIT* among 5 CC subtypes. **** represents *P* value <.0001. CC = cervical cancer, CD = cluster of differentiation, ESTIMATE = Estimation of STromal and Immune cells in MAlignant Tumors using Expression data, NK = natural killer, TPM = transcripts per million.

### 3.4. Differences in drug sensitivity among TCGA-CESC subtypes

Next, we assessed the differences in drug sensitivity among CC patients from different subtypes using the “oncoPredict” package based on the Cancer Therapeutics Response Portal version 2 database. The heatmap of IC50 values for various small-molecule compounds across subtypes is presented in Figure 4A. Given that patients in cluster 2 exhibited poorer prognoses, whereas those in clusters 3 and 4 showed better outcomes, we defined cluster 2 as the poor prognosis group and clusters 3 and 4 as the better prognosis group. Following this, we compared the IC50 values of small-molecule compounds between these 2 groups. The analysis revealed that VX-680, CIL70, and BRD-K01737880 had significantly lower IC50 values in the poor prognosis group than in the better prognosis group, indicating that patients with unfavorable prognoses were more sensitive to these 3 small-molecule compounds (Fig. 4B and C).

**Figure 4.**
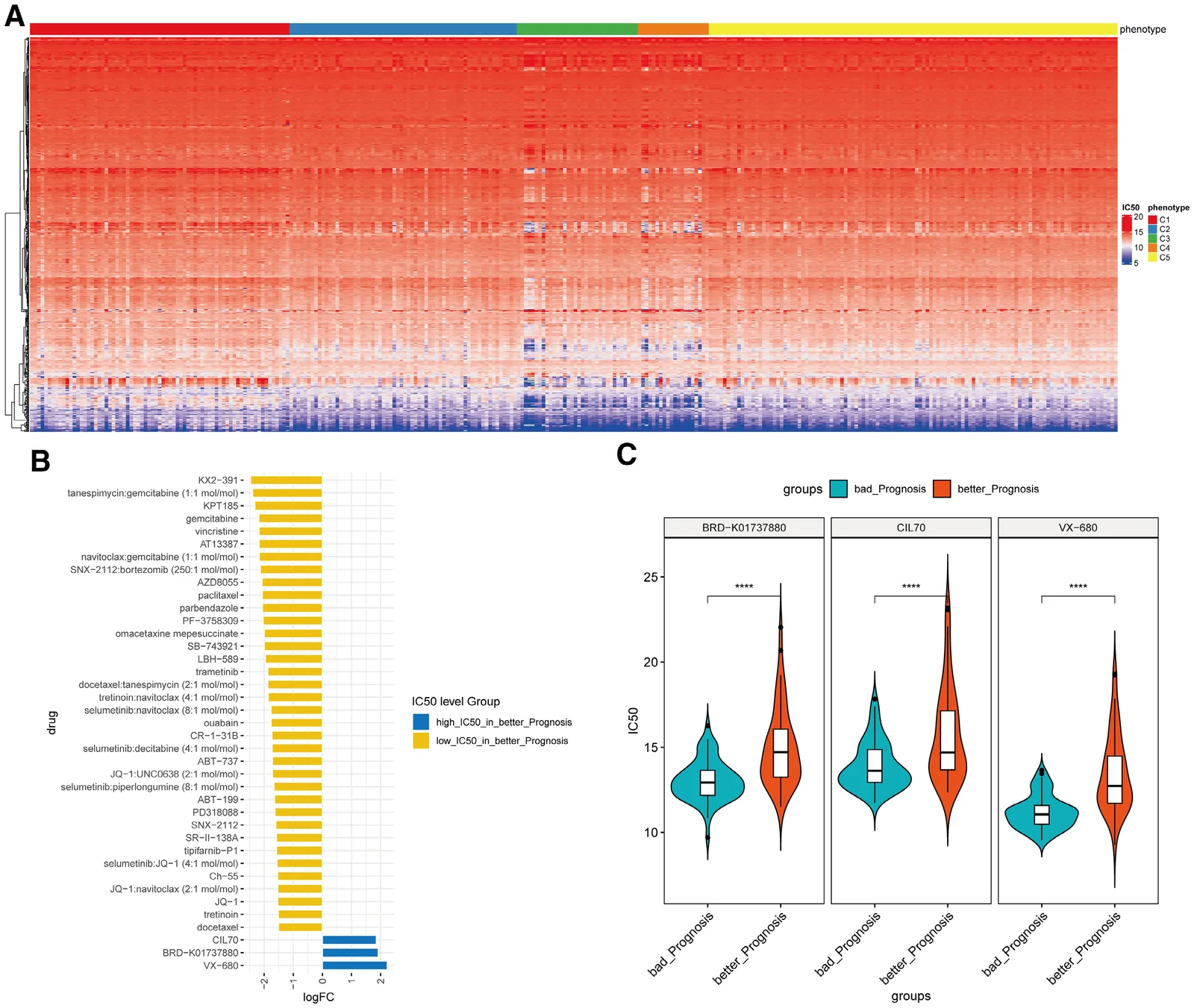
Drug sensitivity analysis among different CC subtypes. (A) The heatmap of the IC50 values of chemotherapy drugs on different CC subtypes. (B) Difference in IC50 values of chemotherapy drugs between bad prognosis and better prognosis groups. (C) The IC50 values of BRD-K01737880, CIL70, and VX-680 between bad prognosis and better prognosis groups. **** represents *P* value <.0001. CC = cervical cancer, IC50 = half-maximal inhibitory concentration.

### 3.5. Identification of core genes in TCGA-CESC subtypes

To characterize subtype-associated expression patterns of nucleotide metabolism-related genes among the 5 CC subtypes, Mfuzz soft clustering was performed using mean gene-expression values arranged according to the original NMF cluster labels (clusters 1–5), resulting in 9 distinct expression-pattern clusters (Fig. 5A). This ordering was used solely as an analytical dimension and did not represent longitudinal sampling or temporal progression. Notably, 7 genes in Mfuzz cluster 4 (*SERPINE1*, *CAV1*, *SOD2*, *SLC16A1*, *XDH*, *CYP27B1*, and *NAGS*) showed the highest expression levels in NMF cluster 2, whereas their expression levels were lower in NMF clusters 3 and 4 (Fig. 5B).

**Figure 5.**
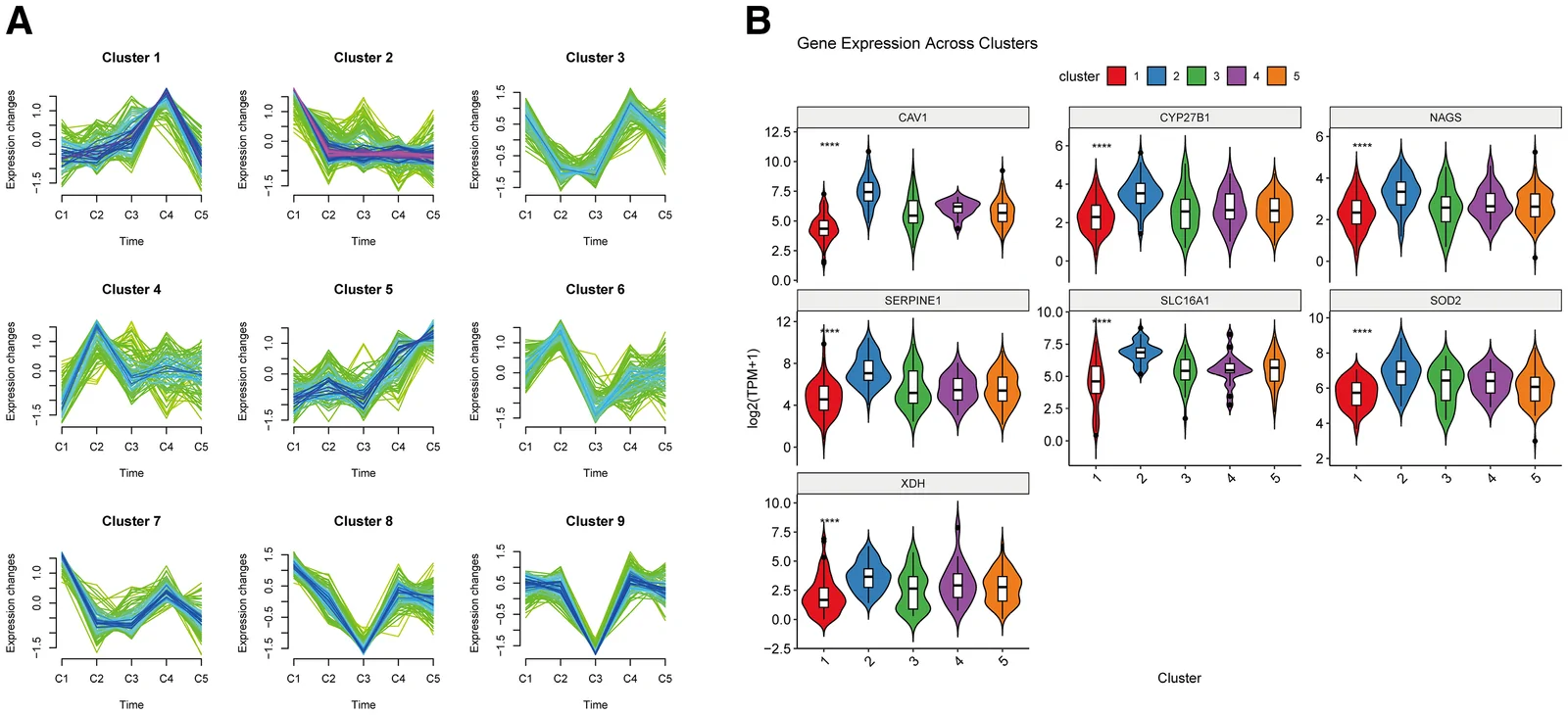
Identification of core genes in CC patients. (A) Mfuzz-based clustering of subtype-associated expression patterns in CC patients. (B) The expression of *CAV1*, *CYP27B1*, *NAGS*, *SERPINE1*, *SLC16A1*, *SOD2*, and *XDH* among 5 CC subtypes. **** represents *P* value <.0001. CC = cervical cancer, TPM = transcripts per million.

### 3.6. Identification of DEGs between high- and low-score groups

Next, GSVA was applied to calculate the scores of *SERPINE1*, *CAV1*, *SOD2*, *SLC16A1*, *XDH*, *CYP27B1*, and *NAGS* for each CC subtype, and the samples were stratified into high- and low-score groups according to the median value (Fig. 6A). Differential expression analysis revealed 42 DEGs between the 2 groups, comprising 18 upregulated and 24 downregulated genes (Fig. 6B).

**Figure 6.**
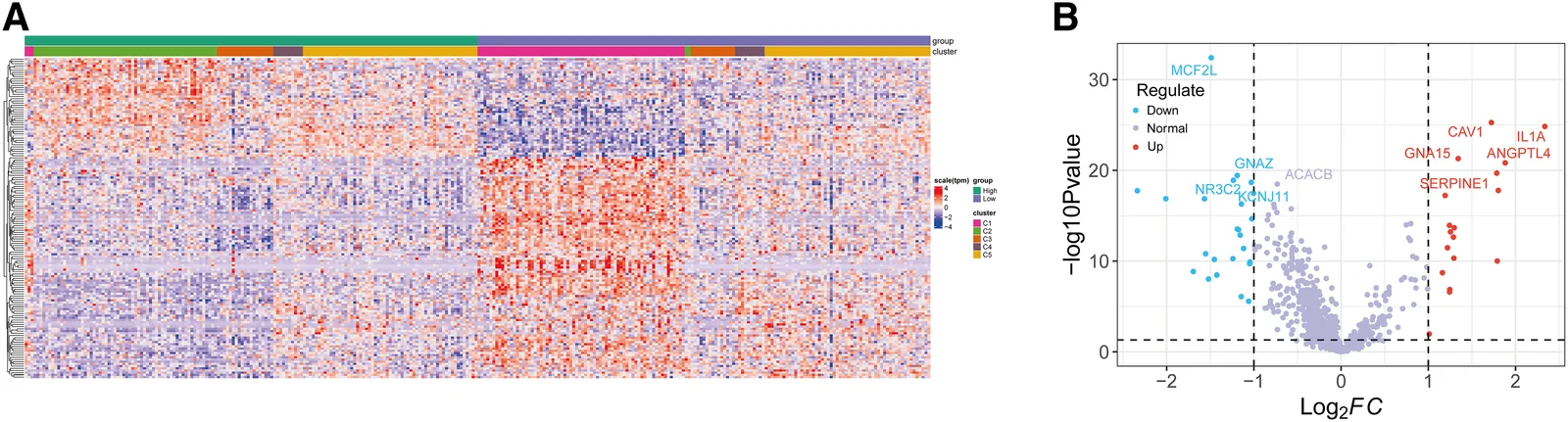
Identification of DEGs between high- and low-score groups. (A) The heatmaps of gene expression profiles in high- and low-group patients. (B) Volcano plot of DEGs in high- and low-group patients. DEGs = differentially expressed genes.

### 3.7. Functional enrichment analysis of DEGs

To elucidate the biological significance of DEGs, GO and KEGG enrichment analyses were performed. GO analysis encompassed 3 categories: biological processes, cellular components, and molecular functions. The results showed that DEGs were mainly enriched in biological processes related to responses to xenobiotic stimuli, nutrient levels, and small-molecule catabolic processes. Cellular components analysis indicated localization to the mitochondrial matrix, the heterotrimeric G-protein complex, and the guanosine triphosphatase complex. Molecular functions analysis revealed enrichment in flavin adenine dinucleotide binding, nicotinamide adenine dinucleotide phosphate binding, and oxidoreductase activity acting on aldehyde or oxo group donors (Fig. 7A). Additionally, KEGG pathway analysis demonstrated that DEGs were predominantly involved in the advanced glycation end product-receptor for advanced glycation end products signaling pathway in diabetic complications; valine, leucine, and isoleucine degradation; and Chagas disease (Fig. 7B). To further explore the pathways most influenced by differential gene expression, GSEA was conducted, identifying significant enrichment in the inflammatory response, tumor necrosis factor signaling pathway, apoptosis, and hypoxia pathways (Fig. 7C).

**Figure 7.**
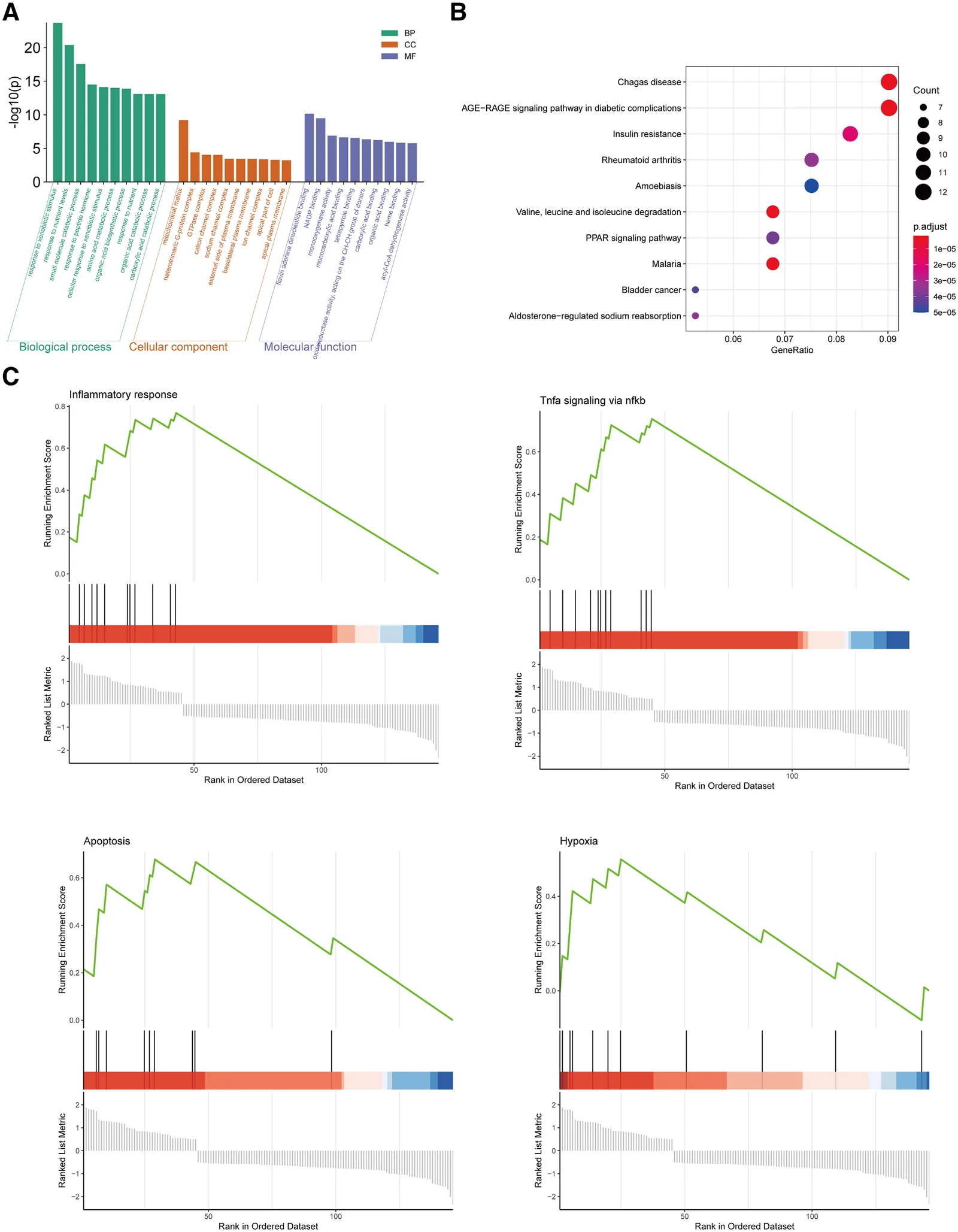
Functional enrichment analysis of DEGs. (A) Barplot of the top 10 most significant GO terms. (B) Dotplot of the top 10 most significant KEGG pathways. (C) GSEA results of inflammatory response, TNF signaling pathway, apoptosis, and hypoxia. AGE-RAGE = advanced glycation end product-receptor for advanced glycation end products, DEGs = differentially expressed genes, GO = Gene Ontology, GSEA = gene set enrichment analysis, KEGG = Kyoto Encyclopedia of Genes and Genomes, PPAR = peroxisome proliferator-activated receptor, TNF = tumor necrosis factor.

### 3.8. Construction of a prognostic model based on core genes in TCGA-CESC

To investigate the association between core genes and the prognosis of CC patients, multiple machine learning algorithms were employed to construct prognostic models including Cox proportional hazards regression with boosting, GBM, partial least squares cox regression, random survival forest, survival support vector machine, elastic net (α = 0.1), lasso, ridge regression, and supervised principal component analysis. The predictive performance of each model was evaluated using the C-index, which ranges from 0 to 1 with higher values indicating superior accuracy. Among these algorithms, GBM achieved the highest C-index and was, therefore, selected as the optimal model for prognostic construction (Fig. 8A and B). Based on the risk scores generated by the GBM model, CC patients were stratified into high- and low-risk groups. Multivariate Cox regression analysis identified the risk score as an independent prognostic factor (Fig. 8C). Kaplan–Meier survival analysis further demonstrated that patients in the high-risk group exhibited significantly shorter overall survival compared with those in the low-risk group (Fig. 8D). Furthermore, time-dependent ROC analysis yielded area under the curve values of 0.62, 0.67, and 0.61 for 1-, 3-, and 5-year survival, respectively, indicating that the model had moderate discriminatory performance and may provide complementary prognostic information (Fig. 8E).

**Figure 8.**
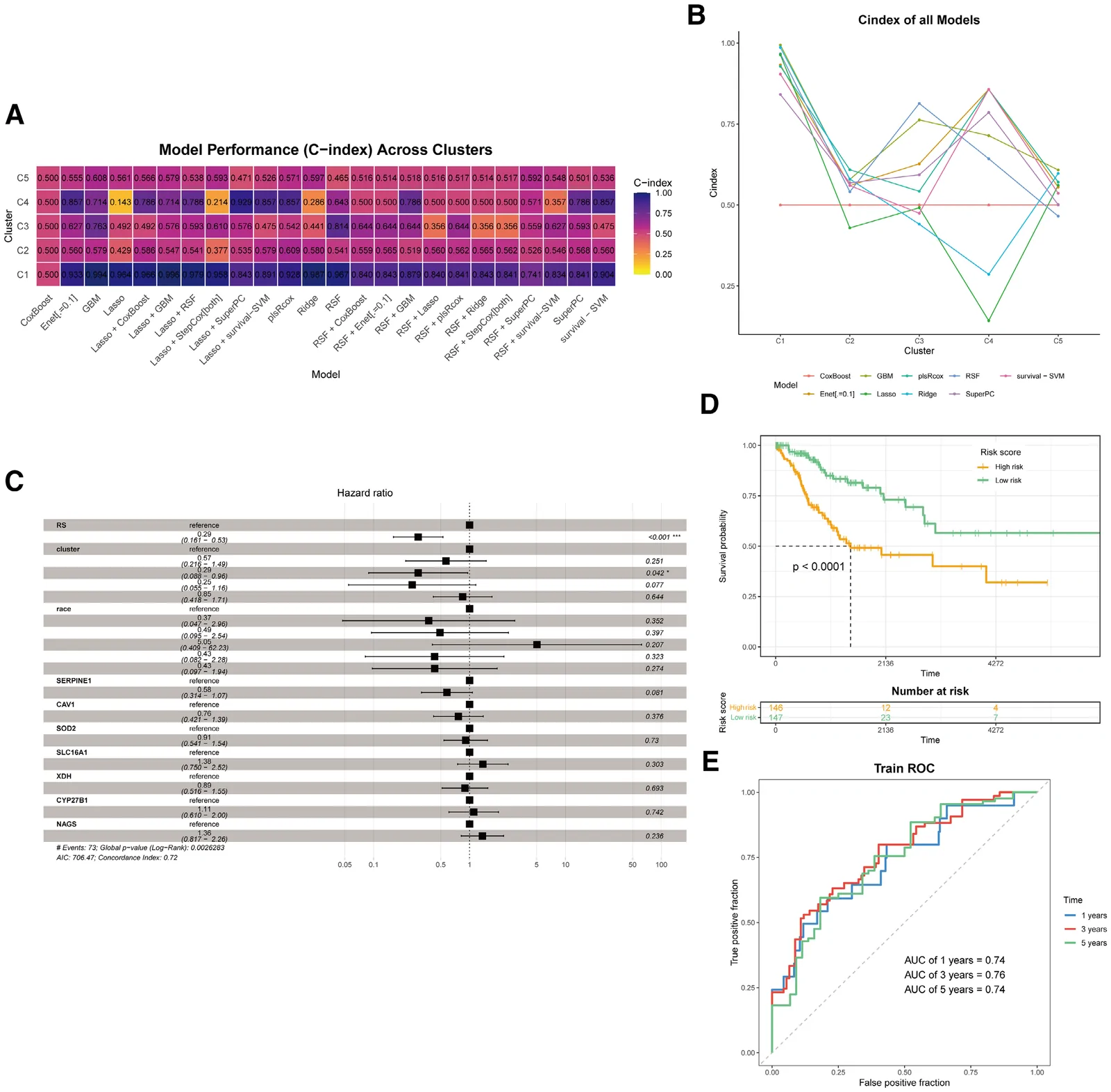
Construction of a prognostic model in CC patients. (A) The heatmap of C-index scores for different machine learning model algorithms. (B) The line chart of C-index scores for different machine learning model algorithms. (C) Forest plot of multivariate Cox regression analysis in CC patients. (D) Kaplan–Meier analysis of overall survival between high-risk and low-risk groups in CC patients. (E) Time-dependent ROC curves for 1-, 3-, and 5-year survival in CC patients. AUC = area under the curve, CC = cervical cancer, CoxBoost = Cox proportional hazards regression with boosting, GBM = generalized boosted regression model, ROC = receiver operating characteristic curve, RSF = random survival forest, SVM = support vector machine, SuperPC = supervised principal component analysis.

### 3.9. Analysis of ICI treatment efficacy in high- and low-risk patient groups

The TIDE framework, which models 2 key mechanisms of tumor immune evasion, was employed to predict the potential efficacy of ICI therapy. The TIDE scores were calculated for patients in the high- and low-risk groups to assess their likely clinical response. The analysis revealed that low-risk patients exhibited higher microsatellite instability and dysfunction scores, whereas TIDE and exclusion scores were lower compared with the high-risk group (Fig. 9A–D).

**Figure 9.**
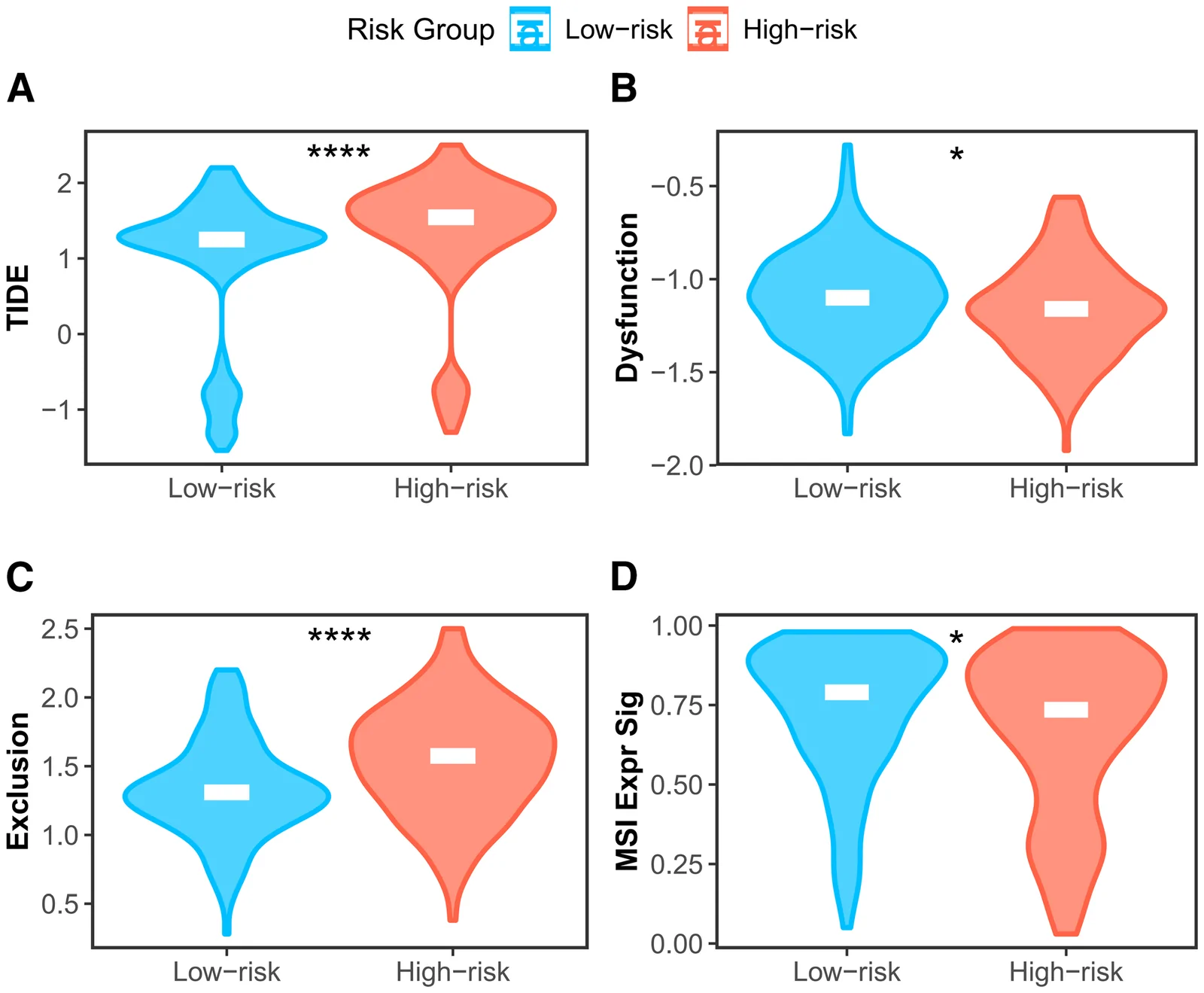
ICIs treatment effect analysis between high- and low-risk groups. (A) Differences in TIDE scores between high- and low-risk groups. (B) Differences in dysfunction scores between high- and low-risk groups. (C) Differences in exclusion scores between high- and low-risk groups. (D) Differences in MSI scores between high- and low-risk groups. ** represents *P* value <.05, **** represents *P* value <.0001. MSI = microsatellite instability, TIDE = tumor immune dysfunction and exclusion.

### 3.10. Molecular docking analysis

Finally, molecular docking was conducted using AutoDock Vina to evaluate the interactions between the 7 core genes and 3 small-molecule compounds (CIL70, BRD-K01737880, and VX-680) that exhibited lower IC50 values in the poor prognosis group. The analysis yielded negative binding-energy estimates for all evaluated compound–protein pairs, indicating energetically favorable docking poses under the applied in silico conditions (Table 1).

**Table 1:** The results of molecular docking for core genes and small-molecule compounds.

Protein	Compound	Binding affinity (kcal/mol)
*CAV1*	CIL70	−6.6
	BRD-K01737880	−7.2
	VX-680	−6.3
*CYP27B1*	CIL70	−9
	BRD-K01737880	−10.5
	VX-680	−9.4
*NAGS*	CIL70	−7.2
	BRD-K01737880	−9.2
	VX-680	−8.7
*SERPINE1*	CIL70	−6.4
	BRD-K01737880	−7.5
	VX-680	−7.7
*SLC16A1*	CIL70	−8.8
	BRD-K01737880	−9.3
	VX-680	−9.3
*SOD2*	CIL70	−5.8
	BRD-K01737880	−6.9
	VX-680	−7.2
*XDH*	CIL70	−7.5
	BRD-K01737880	−9.6
	VX-680	−8.4

CIL70, BRD-K01737880, and VX-680 are small-molecule compound identifiers retrieved from PubChem. *CAV1*, *SERPINE1*, *SOD2*, *SLC16A1*, *XDH*, *CYP27B1*, and *NAGS* denote the gene symbols encoding the core proteins. Binding affinity (kcal/mol) refers to the AutoDock Vina docking score; lower values indicate stronger binding.

## 4. Discussion

CC is a malignant neoplasm that poses a significant threat to women’s health worldwide. It ranks 4th in terms of incidence and mortality among female malignancies globally.^[25–27]^ Identifying biologically relevant molecular subtypes and prognostic biomarkers may therefore improve our understanding of disease heterogeneity and support patient risk stratification. Nucleotide metabolism supplies essential substrates for nucleic acid synthesis and supports the rapid proliferation of tumor cells.^[9]^ Its dysregulation has been implicated in the progression of several cancers. For example, *UHMK1* promotes purine synthesis through *ATF4* activation in gastric cancer,^[28]^ whereas *CLK3* regulates purine metabolism and tumor progression in cholangiocarcinoma.^[29]^ In this study, we acquired gene expression profiles and the corresponding clinical data of CC patients from the TCGA database. Subsequently, we classified CC patients into 5 distinct subtypes based on nucleotide metabolism-related genes using NMF. Survival analysis revealed significant prognostic differences among the subtypes. Specifically, patients classified within cluster 2 exhibited poorer prognoses, whereas those in clusters 3 and 4 demonstrated more favorable outcomes. These findings suggest that the distinct subtypes may be associated with differing pathological mechanisms.

To further elucidate the biological characteristics of the distinct CC subtypes, we conducted somatic mutation profiling across patient groups. Somatic mutations are pivotal in tumor initiation and progression as they alter cellular genetic material, leading to uncontrolled proliferation and malignant transformation.^[30]^ Our analysis revealed a high frequency of somatic mutations among subtypes, suggesting that CC generally exhibits an elevated mutational burden likely linked to genomic instability or DNA repair deficiencies. Among the 3 CC subtypes (clusters 2, 3, and 4), *PIK3CA*, *KMT2C*, *HUWE1*, and *TTN* were the most frequently mutated genes. *PIK3CA*, a critical regulator of the phosphoinositide 3-kinase/protein kinase B signaling pathway, promotes cell growth and survival upon mutation.^[31]^ *KMT2C*, a chromatin-modifying enzyme, influences histone modification and transcriptional activity,^[32]^ whereas *HUWE1*, an E3 ubiquitin ligase, participates in protein degradation control.^[33]^ Although *TTN* is primarily associated with muscle function, its mutations may contribute to tumorigenesis through the disruption of cytoskeletal integrity or as passenger alterations.^[34]^ Furthermore, co-occurrence analysis demonstrated significant co-mutation patterns among recurrently mutated genes, implying that CC pathogenesis involves synergistic effects rather than single-gene alterations. Given the central role of immune cells in CC progression, we next assessed immune cell infiltration across subtypes. The proportions of B cells, Tregs, CD8+ T cells, and M2 macrophages were markedly increased in patients from clusters 3 and 4. B cells are essential immune components that mediate antibody production and modulate immune responses via antigen presentation and cytokine release.^[35]^ Tregs act as potent immunosuppressors that maintain immune tolerance and homeostasis; within the tumor microenvironment, they interact with other immune cells to establish a layered immunosuppressive network.^[36]^ CD8+ T cells, the primary cytotoxic effectors, directly eliminate tumor cells and are crucial for antitumor immunity.^[37]^ Conversely, M2 macrophages, the predominant subtype of tumor-associated macrophages, promote tumor growth and metastasis through the secretion of immunosuppressive factors and the induction of angiogenesis.^[38]^ Collectively, these findings suggest that subtype-specific immune microenvironment remodeling contributes to the heterogeneity observed among CC patients. Additionally, drug sensitivity analysis was performed to identify potential therapeutic agents for each subtype. Considering that cluster 2 patients exhibited poorer prognoses while clusters 3 and 4 showed favorable outcomes, cluster 2 was classified as the poor prognosis group and clusters 3 and 4 as the better prognosis group. Comparative analysis revealed that VX-680, CIL70, and BRD-K01737880 displayed significantly lower IC50 values in the poor prognosis group, suggesting that these small-molecule compounds may represent promising therapeutic candidates for CC patients with unfavorable outcomes.

To further characterize subtype-associated expression patterns, we performed Mfuzz soft clustering of nucleotide metabolism-related genes using mean expression values across the 5 NMF-defined subtypes, which were arranged according to their original cluster labels (clusters 1–5). This ordering served only as an analytical dimension and should not be interpreted as longitudinal sampling or temporal progression. The analysis identified 9 expression-pattern clusters. Genes assigned to Mfuzz cluster 4 showed the highest expression levels in NMF cluster 2, whereas their expression levels were lower in NMF clusters 3 and 4. This subtype-associated expression pattern paralleled the observed prognostic differences, as patients in NMF cluster 2 had poorer outcomes, whereas those in NMF clusters 3 and 4 had more favorable outcomes. Accordingly, 7 genes in Mfuzz cluster 4 (*SERPINE1*, *CAV1*, *SOD2*, *SLC16A1*, *XDH*, *CYP27B1*, and *NAGS*) were selected as core genes potentially associated with unfavorable prognosis in patients with CC. *SERPINE1* encodes a key regulator of the fibrinolytic system that inhibits plasminogen activators, maintaining fibrinolytic balance and preventing excessive bleeding.^[39]^ The equilibrium of the fibrinolytic system is critical for tissue homeostasis and extracellular matrix remodeling processes that are vital for tumor growth, invasion, and metastasis.^[40]^ *CAV1* encodes a principal structural protein of the cell membrane that modulates cell proliferation and survival through regulation of multiple downstream signaling pathways.^[41]^ In glioma, elevated *CAV1* expression has been associated with tumor progression, immune-related features, and resistance to oxidative phosphorylation inhibition.^[42]^ These cross-cancer observations support further investigation of *CAV1* as a potential link between metabolic adaptation and aggressive tumor behavior, although they do not establish the same mechanism in CC. *SOD2*, a mitochondrial antioxidant enzyme, eliminates reactive oxygen species and protects cells from oxidative damage. Oxidative stress is a major driver of CC progression, as it promotes DNA damage, mutagenesis, and aberrant signaling, thereby facilitating tumor initiation and malignant transformation.^[43]^ In tumor tissues, enhanced glycolysis leads to excessive lactate accumulation.^[44]^ *SLC16A1* encodes a key monocarboxylate transporter that mediates lactate and pyruvate transmembrane transport, contributing to the maintenance of metabolic homeostasis in the tumor microenvironment.^[45]^ *XDH*, a critical enzyme in purine metabolism, catalyzes purine oxidation to generate reactive oxygen species, further promoting oxidative stress.^[46]^ *CYP27B1*, a cytochrome P450 enzyme, converts circulating 25-hydroxyvitamin D (25-OHD) into its active form, 1α,25-dihydroxyvitamin D (1,25(OH)_2_D). Active vitamin D binds to the vitamin D receptor to regulate target gene transcription, thereby influencing calcium metabolism, cell growth, and differentiation.^[47]^ NAGS serves as a key enzyme in the urea cycle, catalyzing the synthesis of N-acetylglutamate to regulate ammonia detoxification and nitrogen balance.^[48]^ Disruption of nitrogen metabolism can alter anabolic activity and promote tumor cell proliferation.^[49]^

Next, we applied GSVA to calculate the enrichment scores for the 7 core genes. Based on the median score, CC patients were stratified into high-score and low-score groups, followed by differential expression analysis. Compared with the low-score group, 42 genes exhibited significant alterations in expression in the high-score group. To further characterize the biological roles of these DEGs, GO and KEGG pathway enrichment analyses were performed. The results revealed that these genes were mainly enriched in biological processes related to responses to xenobiotic stimuli, nutrient levels, and small-molecule catabolism and were associated with pathways such as the advanced glycation end product-receptor for advanced glycation end products signaling pathway in diabetic complications; valine, leucine, and isoleucine degradation; and Chagas disease. Moreover, GSEA analysis indicated strong enrichment of pathways involved in inflammatory response, tumor necrosis factor signaling, apoptosis, and hypoxia. A recent review of lung cancer further described how metabolic reprogramming, including alterations in nucleotide metabolism, may contribute to immune resistance by affecting immune-cell function, cytokine signaling, and immune-checkpoint expression.^[50]^ Although these findings were derived from another tumor type, they provide a broader framework for interpreting the potential relationship between nucleotide metabolism and immune regulation observed in CC. Collectively, these findings suggest that the dysregulation of these biological processes and signaling pathways may drive the malignant progression of CC.

To further explore the prognostic relevance of the core genes in CC, we developed prediction models for the 5 CC subtypes using the expression profiles of the 7 core genes across 9 machine learning approaches. Among these models, the GBM algorithm-based model achieved the highest C-index. The C-index is a statistical metric commonly used in clinical studies to assess the predictive accuracy of survival models, with values approaching 1 reflecting superior predictive accuracy.^[51]^ Based on the median risk score generated by the GBM model, patients were stratified into high- and low-risk groups. Multivariate Cox regression identified the risk score as an independent prognostic variable, and Kaplan–Meier analysis showed significantly shorter overall survival in the high-risk group. However, the area under the curve values of 0.62, 0.67, and 0.61 for 1-, 3-, and 5-year survival, respectively, indicated moderate discriminatory performance. Therefore, the model may provide complementary prognostic information but is not sufficient for independent clinical decision-making. Further validation in external cohorts is required to assess its generalizability and potential clinical value.

ICIs have demonstrated therapeutic efficacy across various malignancies.^[52]^ Accordingly, we assessed the potential response to ICI therapy in CC patients classified into high- and low-risk groups. Consistent with expectations, individuals in the high-risk cohort exhibited reduced sensitivity to ICI treatment. Similarly, recent transcriptomic and single-cell analyses identified *TMEM71* as an immunomodulatory biomarker with potential value for predicting immune-checkpoint blockade response in breast cancer.^[53]^ This cross-cancer evidence highlights the potential value of incorporating immune-related biomarkers into computational models of immunotherapy response, although it does not directly validate the present TIDE-based predictions in CC.

In addition, molecular docking was performed to explore potential interactions between the proteins encoded by the 7 core genes and 3 candidate compounds (CIL70, BRD-K01737880, and VX-680). The analysis predicted energetically favorable docking poses for the evaluated compound–protein pairs. However, these computational findings are exploratory and should be interpreted as hypotheses rather than evidence of confirmed drug–target interactions. They do not demonstrate direct binding, target specificity, biological activity, or therapeutic efficacy in CC.

This study has several limitations. First, the analyses were primarily based on a single TCGA-CESC cohort, and the lack of external validation limits the generalizability of the findings. Future studies should validate the identified molecular subtypes and the GBM-based prognostic model in independent Gene Expression Omnibus datasets and prospectively collected multicenter cohorts, with further assessment of model discrimination, calibration, and clinical utility. Second, the bioinformatics findings require experimental verification. The expression of the 7 core genes should be examined using quantitative real-time polymerase chain reaction, Western blot, or immunohistochemistry in independent clinical samples. Gene knockdown or overexpression experiments in CC cell lines could further clarify their roles in tumor proliferation, migration, metabolic regulation, and immune-related processes. Third, the TIDE results represent computational predictions rather than observed responses to ICI therapy and should, therefore, be validated in independent cohorts of patients with CC receiving immunotherapy. The clinical translation of ICI-response biomarkers should also consider treatment safety and immune-related adverse events. A bibliometric analysis of ICI-related complications in melanoma demonstrated increasing research attention to this issue.^[54]^ In addition, the predicted drug sensitivities and molecular docking results are exploratory and require confirmation through biochemical binding assays and cellular experiments. Finally, the relatively limited sample size and moderate discriminatory performance of the prognostic model restrict its current clinical applicability.

## 5. Conclusion

In conclusion, this study identified nucleotide metabolism-related molecular subtypes and candidate biomarkers in the TCGA-CESC cohort. Mfuzz-based clustering of subtype-associated expression patterns further identified 7 core genes potentially associated with prognosis. The GBM-based model showed moderate discriminatory performance in the internal assessment and may provide complementary prognostic information. In addition, the drug sensitivity and molecular docking analyses generated candidate compounds and drug–target interaction hypotheses that warrant further experimental investigation. Collectively, our findings highlight novel biomarkers and potential therapeutic targets for CC, providing important insights for prognostic assessment in CC.

## Acknowledgments

The authors thank the public database TCGA. During the preparation of this manuscript, the authors used ChatGPT (OpenAI) exclusively for the purpose of language polishing and refinement, including rephrasing sentences to improve the clarity, fluency, and grammatical accuracy of the English text and reviewing the wording of specific paragraphs of the Introduction and Discussion sections. The tool was not used to generate or analyze research data, create images, figures, or graphical elements, formulate research hypotheses, interpret results, or draw scientific conclusions, and it was not used to write the initial draft of the manuscript; all scientific content was created by the authors. The authors have thoroughly reviewed and verified the entire content of the manuscript and take full responsibility for all data, analyses, interpretations, and conclusions. All statistical analyses reported in this study were designed and performed by the authors, who have the methodological expertise in bioinformatics and statistical analysis required for this work, using established analytical methods and standard software (R version 4.2.0). No external statistical consultation was obtained, and the authors take full responsibility for the statistical content of the manuscript.

## Author contributions

**Conceptualization:** Feng Li.

**Data curation:** Chaoen Bao.

**Formal analysis:** Chaoen Bao.

**Investigation:** Yinliang Liu.

**Project administration:** Bing Liu.

**Software:** Yinliang Liu.

**Writing – original draft:** Feng Li.

**Writing – review & editing:** Fushan Zhai.
